# The Recurring Word in the Scientific Articles about the Role of Mg in Living Systems Is “Key”

**DOI:** 10.3390/ijms241210100

**Published:** 2023-06-14

**Authors:** Jeanette A. Maier, Stefano Iotti

**Affiliations:** 1 Department of Biomedical and Clinical Sciences, Università di Milano, 20157 Milano, Italy; jeanette.maier@unimi.it; 2 Department of Pharmacy and Biotechnology, University of Bologna, 40127 Bologna, Italy; 3 National Institute of Biostructures and Biosystems, 00136 Rome, Italy

Magnesium (Mg) is a versatile element involved in all aspects of life on our planet. In plants, beyond being the core of chlorophyll, Mg is central to many biochemical and physiological processes. Animals need Mg for their metabolic pathways and various physiological functions. Importantly, animals and plants share the feature of storing energy as Mg-ATP, the driving force of development, differentiation, and growth [1]. Therefore, there is no doubt about the “key” role of Mg in shaping life on Earth. Novel perspectives about the function of Mg emerge in the article by Ohyama [2], who shows that Mg^2+^ influences the higher-order structures of chromatin and chromosomes, and the mechanisms underlying phase separation, including the heterochromatin domain formation. Mg^2+^ seems to be a key regulator of chromatin dynamics and chromatin-based biological processes, thus substantiating previous data linking Mg to epigenetics. The question that arises is how the local Mg^2+^ concentration levels are regulated in the nucleus. By acting in the cytosol, in the nucleus, and in various subcellular organelles, Mg carves cell differentiation in a complex and cell-specific fashion. Although the cellular and molecular mechanisms are not completely unveiled, Mg is pivotal in osteogenic differentiation. The study by Picone et al. [3] provides the first experimental evidence about the presence of Mg in the mineral deposits generated during biomineralization in human osteosarcoma cells, suggesting that Mg incorporation occurs in the early phases of the bone-forming process. However, an intriguing question arises: is the increase of intracellular Mg content in differentiating cells a typical feature of osteogenic differentiation or a peculiarity of osteosarcoma cells? While this study attests to the involvement of Mg in biomineralization, further investigations in primary bone cells are needed. That Mg is an important player in bone is highlighted by the link between low Mg levels and osteopenia [1], whereas little is known about the effects of a high concentration of this element on bone cells. “Dosis sola facit venenum-The dose makes the poison”, wrote Paracelso nearly five centuries ago. This is the case for Mg in the bone. Mammoli et al. [4] demonstrate that high Mg levels are detrimental to bone not only through the inhibition of the osteogenic differentiation of osteoblasts’ precursors but also through the simultaneous increase of osteoclasts’ activity. This study was carried out using a long-lasting treatment with supra-physiological Mg concentrations. Therefore, it will be of great interest to conduct a similar study by exposing cultured bone cells to a transient stimulation with Mg. The emerging hypothesis is that Mg might reprogram vitamin D3 activity on bone remodeling, causing an unbalance between osteoclasts and osteoblasts. As reported by Yamanaka [5], Mg is critical also in neuronal development and alterations of its homeostasis contribute to neuroinflammation and neurodegeneration. Accordingly, appropriate Mg^2+^ supplementation exhibits neurotrophic effects in Parkinson’s disease, Alzheimer’s disease, and demyelination. It is feasible that recovery of healthy Mg^2+^ homeostasis through chemotherapy targeting Mg^2+^-transporting systems can improve neuronal and glial functions under pathological conditions. Innovative experimental approaches—such as brain organoids—might help to deepen present knowledge about Mg in neuro(patho)physiology [6] and develop new diagnostic tools and therapies. Regarding Parkinson’s disease (PD), interesting hints derive from the study by Cibulka et al. [7], who focus their interest on the gene SLC41A1, which is localized within PD-susceptibility locus PARK16 and encodes for a Na^+^/Mg^2+^-exchanger. The aim is to investigate the PD diagnostic potential of several SLC41A1 single nucleotide polymorphisms (SNP) in the Slovak population by using random-forest machine-learning algorithms trained in four various modes. The results further corroborate the evidence about differences between diverse populations regarding the association of SLC41A1 polymorphisms with PD susceptibility. Mg is also implicated in the maintenance of the highly differentiated phenotype and function of vascular smooth muscle cells [8], pivotal in the maintenance of vascular structures and the control of blood pressure. Moreover, Mg relaxes vascular smooth muscle mainly by competing with Ca. As shown by Chang et al. [9], Mg attenuates the progression of monocrotaline-induced pulmonary hypertension by antagonizing Ca, preventing oxidative stress, mitochondrial injury, and inflammation. These data were recently broadened by a report demonstrating that Mg supplementation mitigates pulmonary hypertension by regulating Mg transporters [10]. Indeed, Mg homeostasis is orchestrated by the interplay of various Mg^2+^ channels and transporters [11]. Among these, TRPM6 and 7 are prominent in coordinating Mg cellular influx. As summarized by Zou [12], they are unique proteins characterized by their channel and kinase domains, which differentially regulate cell signaling and function. TRPM6 is mostly expressed in epithelial cells of the kidney and gastrointestinal system, whereas TRPM7 is ubiquitously expressed. TRPM6 and cellular Mg^2+^ homeostasis in the kidney is regulated by epidermal growth factor (EGF) signaling through its receptor (EGFR). Inhibition of EGFR signaling impairs TRPM6 function leading to renal Mg^2+^ wasting and consequent hypomagnesemia in cancer patients treated with EGFR inhibitors. Changes in TRPM7 activity and altered Mg^2+^ homeostasis significantly affect tyrosine kinase signaling. Hence, receptor tyrosine kinases (RTKs) influence TRPM7, which may influence tyrosine kinase signaling. The putative crosstalk between TRPM7 and RTKs has clinical relevance because inhibitors of RTKs, including VEGFR and EGFR, are increasingly used to treat cancer and inflammatory diseases. Hence, knowing the mechanisms whereby these drugs cause hypomagnesemia is important to manage unwanted side effects in a mechanism-specific manner. As underscored by Grober, Mg loss and, consequently, hypomagnesemia result from many widely used drugs, such as diuretics and proton-pump inhibitors [13]. The author urges physicians and pharmacists to pay particular attention to individuals under long-term medication with proton pump inhibitors or diuretics who should be monitored for drug-induced magnesium deficiency. Attention should also be devoted to the adverse effects of various therapy on magnesium status to minimize the potential risk to the health, especially in high-risk individuals (e.g., children, the elderly, patients with diabetes, patients with hypertension, and patients on polypharmacotherapy).

While more and more data are accumulating unprecedentedly about the central role of Mg in life sciences, more and more questions are arising and require careful evaluation. What is the best chemical formulation to study in vivo and in vitro the biological and biochemical function of Mg? Which is the most accurate method to measure intracellular Mg? Is it more reliable to evaluate total or free Mg? Despite thousands of publications, there are still more questions than answers. Yet, this is what science is: asking better the right questions.
